# Incorporate delivery, warming and washing methods into efficient cryopreservation

**DOI:** 10.3389/fbioe.2023.1215591

**Published:** 2023-06-15

**Authors:** Wenqian Zhang, Xiangjian Liu, Yuying Hu, Songwen Tan

**Affiliations:** Xiangya School of Pharmaceutical Sciences, Central South University, Changsha, Hunan, China

**Keywords:** cryopreservation, DMSO, cryoprotectant delivery, novel warming, automatic washing

## 1 Introduction

Cryopreservation is a popular and crucial method for long-term storage of cells, tissues, and other biological samples at low temperatures. During this process, the cells are in a state of “suspended animation” to inhibit biological and chemical reactions (Pegg, 2015; Jang et al., 2017; Chang and Zhao, 2021). Recently, there are two main strategies of cryopreservation: slow freezing and vitrification (Kometas et al., 2021). Slow freezing refers to the freezing of biological samples at a rate of 1°C/min. This can be achieved through laboratory freezing tubes and programmed cooling boxes (Garcia-Flores et al., 2023). Vitrification means that when a small biological sample cools at a very fast rate, the internal water will be transformed into a glassy state (Schulz et al., 2020). The devices for vitrification are various, such as cyrotop (for oocytes) (Miao et al., 2022), plastic straw (for spermatids) (Patra et al., 2021), and cryomesh (for islets) (Zhan, Rao, et al., 2022). However, low temperature can cause a range of damage to biological samples, including protein denaturation (Chen et al., 2022), membrane damage (Lee et al., 2023), oxidative stress (Gualtieri et al., 2021). Since DMSO was first used in bull sperm cryopreservation in 1959, it has been found that the addition of a certain concentration of DMSO could resist these cryodamages (Lovelock and Bishop, 1959; Stubbs et al., 2020). Unfortunately, DMSO can lead to various problems such as differentiation of human stem cells (Davidson et al., 2015), hemolysis (Yi et al., 2017), and alterations in DNA methylation (Verheijen et al., 2019) at body temperature (37°C).Therefore, a series of novel CPAs, such as AFP, proline, etc., have been developed for DMSO-free cryopreservation (Li et al., 2020; Weng and Beauchesne, 2020), and these CPAs can be classified as permeable or impermeable according to whether they can enter cells (Weng et al., 2019; Yong et al., 2020). But none of them can replace DMSO totally. Currently, the most common cryopreservation process involves three steps: 1) mixing DMSO with biological samples and storing them at low temperature; 2) thawing by convection rewarming; and 3) removing DMSO by centrifugation and washing (Jang et al., 2017; Whaley et al., 2021). Although this protocol is widely used in clinics and laboratories, there are still some challenges.

Besides the toxicity of DMSO, commonly used convective rewarming can lead to ice recrystallization and devitrification because of its slow rewarming rates (Marquez-Curtis et al., 2015; Wang et al., 2016). Also, uneven temperature distribution and thermal gradients can induce thermal stress and destroy the biological samples, especially for larger volumes (Taylor et al., 2019). Finally, the manual centrifugation and washing to remove CPA is not only demanding for operators, but may also lead to complex procedures and unintended cell loss (Shu et al., 2014; Hornberger et al., 2019). In general, these procedures may cause damage instead of thoroughly cleaning (Fois et al., 2007).

**FIGURE 1:**
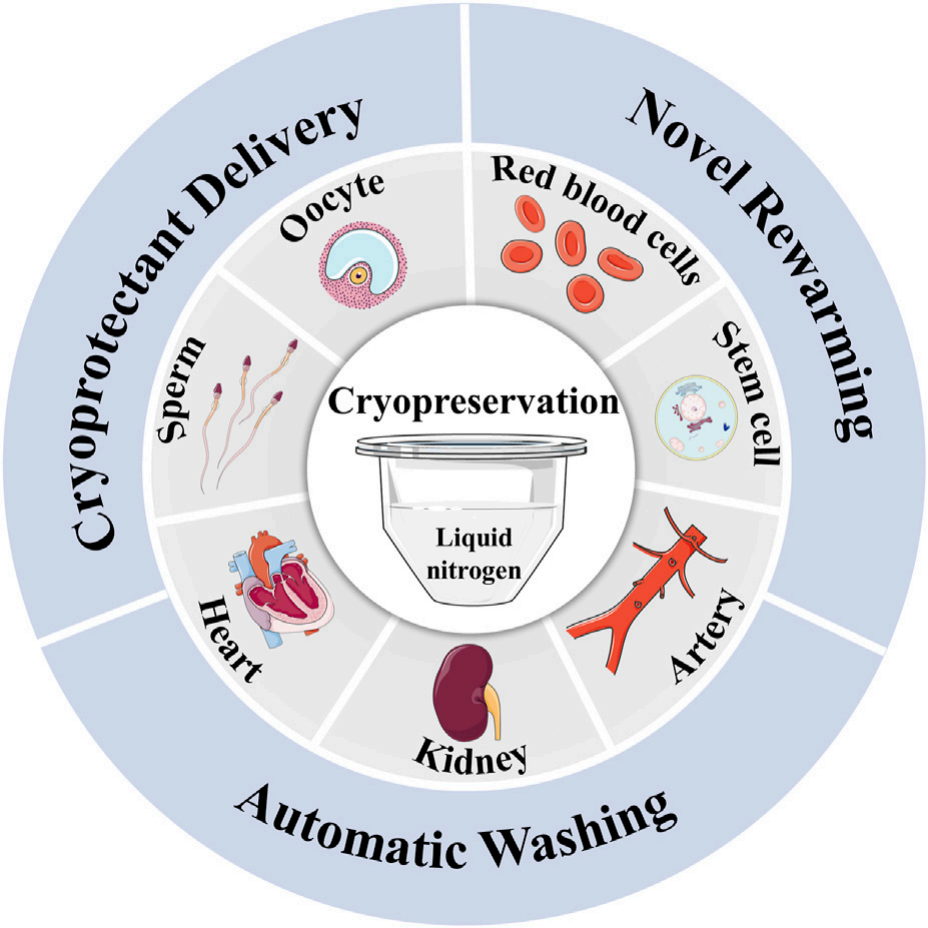
The novel delivery, warming and washing methods for cryopreservation of biological samples.

To solve the problems discussed above, advanced cryopreservation technologies must be employed. Initially, impermeable CPA is widely used in cryopreserving biological samples such as oocytes and red blood cells due to its non-toxicity, high efficiency, and stability (Stoll et al., 2012; Zhang et al., 2016; Huang et al., 2017). However, its impermeability hinders its application in preventing intracellular damage. The use of delivery methods like nanoparticles (Rao et al., 2015) and membrane perturbation (Janis et al., 2021) are required to ensure its presence inside or outside the cells. In addition, novel warming methods such as nanowarming offers a faster and more even heating option compared to convective rewarming. It is especially important in cryopreserving large volume biological samples (Manuchehrabadi et al., 2017). Furthermore, high-quality washing methods have become effective way of convenient removal of CPA (Lusianti and Higgins, 2014; Zhao and Fu, 2017). Therefore, the adoption of these advanced cryopreservation technologies provides an opportunity to achieve efficient and high-quality cryopreservation (Figure 1).

## 2 CPA delivery methods

On the one hand, trehalose has the ability to form hydrogen bonds with biomacromolecules and promote hydration, enabling cell components to maintain functional conformations. It can also slow metabolic activity by forming glassy substrates with extremely low molecular mobility (He, 2011; Ntai et al., 2018; Hu et al., 2023). On the other hand, AFP can enhance the resistance of cells to cryoinjury by inhibiting ice crystal growth and interaction with membranes (Kim et al., 2017; Baskaran et al., 2021). Due to these properties, trehalose and AFP have gained attention as non-toxic and reliable CPAs (Lee et al., 2013; Huang et al., 2017). However, unlike DMSO, trehalose and AFP cannot penetrate the cell membrane, which limits their use in protecting cells from intracellular ice crystal damage (Chang and Zhao, 2021; Hu et al., 2023). Therefore, effective methods for delivering trehalose and AFP are essential for successful cryopreservation. These methods include nanoparticles carriers and membrane perturbation delivery, depending on cellular structure and function. It must be noted that trehalose cannot be metabolized in cells and the safety of intracellular AFP is unclear, which may hinder their translation to clinic (Campbell and Brockbank, 2012; Dovgan et al., 2017). Detailed information has been summarized in Table 1.

**TABLE 1:** Examples of CPA delivery methods and warming methods.

Methods			Cell types	Positive effects	References
CPA delivery methods	pH-sensitive systems delivery	GNPs^a^	hADSCs^b^	Cell viability↑	Rao et al. (2015)
				Functional survival↑	
				Attachment efficiency↑	
				Proliferative potential↑	
		CS^c^–TPP^d^ nanoparticles	NK cells^e^	Cell viability↑	Yao et al. (2020)
				Survival rate↑	
				Cytotoxicity↑	
				Proliferative potential↑	
	Cold-responsive systems delivery	PNP^f^	MDA-MB-231 cancer cells^g^ and hADSCs	Cell viability↑	Zhang et al. (2019)
				Attachment efficiency↑	
				Survival rate↑	
				Proliferative potential↑	
				Functional survival↑	
	Membrane perturbation delivery	Apatite nanoparticles	sRBCs^h^	Survival rate↑	Stefanic et al. (2017)
				Cell viability↑	
		ε-PL^i^ and PVP^j^	hRBCs^k^	Trehalose permeability↑	Liu et al. (2022)
				Survival rate↑	
				The amount of PS^*l*^ exposure↓	
		Sonoporation	hRBCs	Cell recovery rate↑	Janis et al. (2021)
				Trehalose permeability↑	
		Ultrasound-integrated PDMS^m^-based microfluidic	hRBCs	Trehalose permeability↑	Centner et al. (2020)
				Cell viability↑	
		Glycopeptide of saccharide-grafted ε-poly (L-lysine)	hRBCs	Survival rate↑ hemolysis↓	Gao et al. (2022)
	Other delivery methods	PAK_T_^n^	MSCs^o^	Cell viability↑	Piao et al. (2022)
				Proliferative potential↑	
				Functional survival↑	
				Ice Recrystallization↓	
		EGFP-ApAFP752^*p*^	HEK 293T^q^	Survival rate↑	Sreter et al. (2022)
				Intracellular AFP concentration↑	
Warming methods	Magnetoresponsive induction heating	msIONPs^r^	HDFs^*s*^, porcine carotid arteries, porcine heart valve leaflet, porcine femoral arteries	Cell viability↑	Manuchehrabadi et al. (2017)
				Survival rate↑	
				Damaged cells↓	
		sIONPs^t^	Rabbit kidney	Tissue integrity↑	Sharma et al. (2021)
				Tissue viability↑	
	Photoresponsive induction heating	Gold nanorods and pulsed lasers	Zebrafish embryos	Functional integrity↑	Khosla et al. (2020)
				Survival rate↑	
		2D-GO-MoS_2_ NSs^u^	HUVECs^v^	Warming rate↑	Panhwar et al. (2018)
				Cell viability↑	
				Survival rate↑	
				Functional integrity↑	
	Photo- and magnetoresponsive induction heating	Graphene Oxide-Fe_3_O_4_ Nanocomposite	MSCs	Proliferative potential↑	Cao et al. (2019)
				Cell viability↑	
				Functional integrity↑	
	Rapid joule heating	Electrical conductor and voltage pulse generator	Adherent fibroblast cells, *Drosophila* embryos and rat kidney slices	Survival rate↑	Zhan, Han, et al. (2022)
				The structural integrity of the kidney slices↑	
	Radiofrequency Heated Metal Forms	Al foil metal	2 mm thick porcine aortas	Tissue viability↑	Han et al. (2020)
	Infrared radiation heating	Focused halogen IR lamp	Heterogeneous human epithelial Caco2^w^ and RPE^x^ cell lines	Cell viability↑	Bissoyi and Braslavsky (2021)
				Cell adhesion ↑	

^a^ GNPs: genipin-cross-linked Pluronic F127-chitosan nanoparticles.

^b^ hADSCs: human adipose-derived stem cells.

^c^ CS: chitosan.

^d^ TPP: tripolyphosphate.

^e^ NK cells: natural killer cells.

^f^ PNP: PLGA (poly (lactic-co-glycolic acid))—pNIPAM-B (poly (N-isopropylacrylamide-co-butyl acrylate)) —PF127(Pluronic F127).

^g^ MDA-MB-231 cancer cells: human breast cancer cells.

^h^ sRBCs: sheep red blood cells.

^i^ ε-PL: ε-poly (L-lysine).

^j^ PVP: poly (vinyl pyrrolidone).

^k^ hRBCs: human red blood cells.

^l^ PS: protoplasmic surface.

^m^ PDMS: polydimethylsiloxane^*n*^PAKT:poly (l-alanine-co-l-lysine)-graft-trehalose.

^o^ MSCs: mesenchymal stem cells^*p*^EGFP-ApAFP752: enhanced green fluorescent protein (EGFP) -tagged antifreeze protein.

^q^ HEK 293T: human embryonic kidney cell line.

^r^ msIONPs: mesoporous silica coated iron oxide nanoparticles ^*s*^HDFs: human dermal fibroblasts.

^t^ sIONPs: silica-coated iron oxide nanoparticles.

^u^ 2D-GO-MoS2 NSs: Two-dimensional graphene oxide molybdenum disulfide nanosheets.

^v^ HUVECs: human umbilical vein endothelial cells.

^w^ Caco2: colorectal adenocarcinoma.

^x^ RPE: retinal pigment epithelium.

### 2.1 Nanoparticles carriers

Endocytosis is one of the mechanisms by which nanoparticles (NPs) can deliver trehalose into cells (Stewart and He, 2019). For instance, cold-responsive nanocapsules (CR-NCs) encapsulated trehalose by microfluidics have successfully maintained the glucose-regulating function of pancreatic β cells after cryopreservation (Cheng et al., 2019). Some pH-responsive delivery systems, such as genipin-cross-linked Pluronic F127-chitosan nanoparticles (GNPs) (Rao et al., 2015) and chitosan-tripolyphosphate (CS-TPP) nanoparticles (Yao et al., 2020) have also shown efficient intracellular delivery of trehalose. Remarkably, Poly (l-alanine-co-l-lysine)-graft-trehalose (PAK_T_) was synthesized as a natural antifreezing glycopolypeptide (AFGP). It can be used as a carrier for trehalose delivery while also mimicking a CPA to inhibit ice recrystallization and protecting cells (Piao et al., 2022).

### 2.2 Membrane perturbation delivery

Membrane perturbation is another method to deliver impermeable CPAs into cells. The effectiveness of this approach has been demonstrated by the delivery of AFPIII via the location of cell-penetrating peptide pep-1 (Tomás et al., 2019). In non-endocytic human red blood cells, trehalose can be delivered by altering membrane permeability, which depends on the interaction between the polymer attached to the hydrophobic side group and the membrane lipid bilayer (Liu et al., 2022). Phenethylamine-grafted PGA (PGA-g-PEA) synthesized from hydrophobic PEA-modified PGA enhances trehalose loading capacity and reduce hemolysis of red blood cells by self-forming nanoparticles in a phosphate buffer solution (Zhang et al., 2020). Besides nanoparticles, ultrasound and microbubbles can also induce transient perforations to achieve trehalose loading into human red blood cells (Janis et al., 2021).

## 3 Warming methods

Convective rewarming, which means immersing biological samples in a water bath heated to 37°C, is still considered the gold standard for rewarming (Wolkers and Oldenhof, 2015). However, the slow heating rate resulting from convective rewarming can lead to ice recrystallization and devitrification (Wang et al., 2016). Additionally, convective rewarming may not provide even heating, particularly as the volume of biological samples increases. The resulting thermal gradients can cause biological samples to crack (Mfarrej et al., 2017; Sharma et al., 2021; Sharma et al., 2023). To overcome these limitations, a sequence of methods for rapid and even rewarming were developed, such as nanowarming, rapid joule heating (Zhan, Han, et al., 2022), infrared radiation heating (Bissoyi and Braslavsky, 2021). These methods have been summarized in Table 1.

### 3.1 Magnetoresponsive induction heating

Néel and Brownian relaxations caused by magnetic moment oscillation can induce the thermal effect of magnetic nanoparticle under an alternating magnetic field (AMF) (Syme et al., 2004; Cazares-Cortes et al., 2017). Therefore, the addition of magnetic nanoparticles to the cryoprotectant solution under an AMF can improve the thermal conductivity of biological samples, resulting in relatively even and rapid heating. This method minimizes damage to biological samples caused by slow and uneven rewarming (Etheridge et al., 2014; Liu et al., 2018). The vitrified organs, including rat hearts (Joshi et al., 2022) and rabbit kidneys (Sharma et al., 2021), have been successfully rewarmed utilizing magnetic iron oxide nanoparticles (IONPs), and the integrity of their structure and function is maintained. But the potential cytotoxicity of nanoparticles must be considered. However, due to the limitation of warming rate, the application of magnetically responsive nanoheating requires high molarity CPA, which brings potential toxicity to biological samples.

### 3.2 Photoresponsive induction heating

Gold nanorods and carbon black micron-sized particles have also been utilized in rewarming methods to achieve photoresponsive inducing heating (Khosla et al., 2020). Laser vibration in the gold nanoparticles induces heat dissipation. This enables ultra-rapid rewarming of cryopreserved zebrafish embryos and improves embryo survival. However, physical damage from injection site increased the probability of ice formation during rapid freezing (Khosla et al., 2019; Khosla et al., 2020). Carbon black micron-sized particles can suddenly heat up and emit heat after absorbing laser infrared energy. This hate will be transferred to the biological sample through the solution to achieve rapid and even heating (Panhwar et al., 2018).Nonetheless, photoresponsive nanoheating is difficult to apply to large-scale biological samples (Zhan, Han, et al., 2022).

## 4 Washing methods

Currently, the removal of DMSO from biological samples still relies on manual centrifugation, which requires skilled operators to remove the supernatant and replace it with a washing solution (Shu et al., 2014). However, cell loss is unavoidable during centrifugation, and residual DMSO can be highly toxic (Syme et al., 2004). Fortunately, several techniques for DMSO removal have been developed to address these challenges.

The hollow fiber module with semi-permeable membrane uses the pressure and concentration difference between the cell membrane and the fiber membrane to remove CPA from cells. This technique can also be scaled up for large cryopreserved cell suspensions (Ding et al., 2010). Dual-flow microfluidic devices have been specifically designed to remove intracellular DMSO in a limited time, which is essential for clinical applications (Fleming Glass et al., 2008). Dilution filtration system has been demonstrated to be more efficient and cost-effective than conventional multistep centrifugation or automated centrifugation (Zhou et al., 2011). Sepax-2 and Lovo devices have also been proven effective in removing DMSO from thawed hematopoietic progenitor cells (HPC), while maintaining the viability of CD34 cells before clinical infusion. However, the washing scheme must be flexible, convenient and low-cost for more common applications (Abonnenc et al., 2017; Mfarrej et al., 2017).

## 5 Conclusion

With the advancement of modern biotechnology, conventional cryopreservation obviously failed to keep pace with current needs. This review generalized the recent advances of delivery, warming and washing methods used in cryopreservation. Delivery methods helped to overcome the major limitation of the ultra-low permeability of impermeable CPAs, enabling their intracellular and extracellular cryopreservation. The use of novel warming methods can achieve rapid and even rewarming while avoiding the adverse effects of devitrification on biological samples. The emergence of various washing methods created a novel platform for convenient and efficient CPA removal. It must be noted that all the novel methods for cryopreservation have not been widely used neither in laboratory nor in clinic due to the high cost and complex operation protocol. Future studies need to focus on making new methods less difficult to perform without reducing their effectiveness, so that they can be applied by more researchers.

## References

[B1] Abonnenc M. Pesse B. Tissot J. D. Barelli S. Lion N. (2017). Automatic washing of thawed haematopoietic progenitor cell grafts: A preclinical evaluation. Vox Sang. 112 (4), 367–378. 10.1111/vox.12503 28337763

[B2] Baskaran A. Kaari M. Venugopal G. Manikkam R. Joseph J. Bhaskar P. V. (2021). Anti freeze proteins (Afp): Properties, sources and applications - a review. Int. J. Biol. Macromol. 189, 292–305. 10.1016/j.ijbiomac.2021.08.105 34419548

[B3] Bissoyi A. Braslavsky I. (2021). Adherent cell thawing by infrared radiation. Cryobiology 103, 129–140. 10.1016/j.cryobiol.2021.08.002 34400151

[B4] Campbell L. H. Brockbank K. G. (2012). Culturing with trehalose produces viable endothelial cells after cryopreservation. Cryobiology 64 (3), 240–244. 10.1016/j.cryobiol.2012.02.006 22366172

[B5] Cao Y. Hassan M. Cheng Y. Chen Z. Wang M. Zhang X. (2019). Multifunctional photo- and magnetoresponsive graphene oxide-Fe(3)O(4) nanocomposite-alginate hydrogel platform for ice recrystallization inhibition. ACS Appl. Mater Interfaces 11 (13), 12379–12388. 10.1021/acsami.9b02887 30865418

[B6] Cazares-Cortes E. Espinosa A. Guigner J. M. Michel A. Griffete N. Wilhelm C. (2017). Doxorubicin intracellular remote release from biocompatible oligo(ethylene glycol) methyl ether methacrylate-based magnetic nanogels triggered by magnetic hyperthermia. ACS Appl. Mater Interfaces 9 (31), 25775–25788. 10.1021/acsami.7b06553 28723064

[B7] Centner C. S. Murphy E. M. Priddy M. C. Moore J. T. Janis B. R. Menze M. A. (2020). Ultrasound-induced molecular delivery to erythrocytes using a microfluidic system. Biomicrofluidics 14 (2), 024114. 10.1063/1.5144617 32341725 PMC7176461

[B8] Chang T. Zhao G. (2021). Ice inhibition for cryopreservation: Materials, strategies, and challenges. Adv. Sci. (Weinh). 8 (6), 2002425. 10.1002/advs.202002425 33747720 PMC7967093

[B9] Chen X. Wu J. Li X. Yang F. Huang D. Huang J. (2022). Snow flea antifreeze peptide for cryopreservation of lactic acid bacteria. NPJ Sci. Food 6 (1), 10. 10.1038/s41538-022-00128-4 35115563 PMC8813996

[B10] Cheng Y. Yu Y. Zhang Y. Zhao G. Zhao Y. (2019). Cold-responsive nanocapsules enable the sole-cryoprotectant-trehalose cryopreservation of beta cell-laden hydrogels for diabetes treatment. Small 15 (50), e1904290. 10.1002/smll.201904290 31595687

[B11] Davidson A. F. Glasscock C. McClanahan D. R. Benson J. D. Higgins A. Z. (2015). Toxicity minimized cryoprotectant addition and removal procedures for adherent endothelial cells. PLoS One 10 (11), e0142828. 10.1371/journal.pone.0142828 26605546 PMC4659675

[B12] Ding W. Zhou X. Heimfeld S. Reems J. A. Gao D. (2010). A steady-state mass transfer model of removing CPAs from cryopreserved blood with hollow fiber modules. J. Biomech. Eng. 132 (1), 011002. 10.1115/1.4000110 20524740 PMC2882658

[B13] Dovgan B. Barlic A. Knezevic M. Miklavcic D. (2017). Cryopreservation of human adipose-derived stem cells in combination with trehalose and reversible electroporation. J. Membr. Biol. 250 (1), 1–9. 10.1007/s00232-016-9916-z 27383230

[B14] Etheridge M. L. Xu Y. Rott L. Choi J. Glasmacher B. Bischof J. C. (2014). RF heating of magnetic nanoparticles improves the thawing of cryopreserved biomaterials. Technology 2 (3), 229–242. 10.1142/s2339547814500204

[B15] Fleming Glass K. K. Longmire E. K. Hubel A. (2008). Optimization of a microfluidic device for diffusion-based extraction of dmso from a cell suspension. Int. J. Heat. Mass Transf. 51 (23-24), 5749–5757. 10.1016/j.ijheatmasstransfer.2008.04.018 19884964 PMC2621076

[B16] Fois E. Desmartin M. Benhamida S. Xavier F. Vanneaux V. Rea D. (2007). Recovery, viability and clinical toxicity of thawed and washed haematopoietic progenitor cells: Analysis of 952 autologous peripheral blood stem cell transplantations. Bone Marrow Transpl. 40 (9), 831–835. 10.1038/sj.bmt.1705830 17724443

[B17] Gao S. Zhu K. Zhang Q. Niu Q. Chong J. Ren L. (2022). Development of icephilic ACTIVE glycopeptides for cryopreservation of human erythrocytes. Biomacromolecules 23 (2), 530–542. 10.1021/acs.biomac.1c01372 34965723

[B18] Garcia-Flores V. Xu Y. Pusod E. Romero R. Pique-Regi R. Gomez-Lopez N. (2023). Preparation of single-cell suspensions from the human placenta. Nat. Protoc. 18 (3), 732–754. 10.1038/s41596-022-00772-w 36451054 PMC10355223

[B19] Gualtieri R. Kalthur G. Barbato V. Di Nardo M. Adiga S. K. Talevi R. (2021). Mitochondrial dysfunction and oxidative stress caused by cryopreservation in reproductive cells. Antioxidants (Basel) 10 (3), 337. 10.3390/antiox10030337 33668300 PMC7996228

[B20] Han Z. Sharma A. Gao Z. Carlson T. W. O'Sullivan M. G. Finger E. B. (2020). Diffusion limited cryopreservation of tissue with radiofrequency heated metal forms. Adv. Healthc. Mater 9 (19), e2000796. 10.1002/adhm.202000796 32875732 PMC7879698

[B21] He X. (2011). Thermostability of biological systems: Fundamentals, challenges, and quantification. Open Biomed. Eng. J. 5, 47–73. 10.2174/1874120701105010047 21769301 PMC3137158

[B22] Hornberger K. Yu G. McKenna D. Hubel A. (2019). Cryopreservation of hematopoietic stem cells: Emerging assays, cryoprotectant agents, and technology to improve outcomes. Transfus. Med. Hemotherapy 46 (3), 188–196. 10.1159/000496068 PMC6558318 31244587

[B23] Hu Y. Liu X. Liu F. Xie J. Zhu Q. Tan S. (2023). Trehalose in biomedical cryopreservation-properties, mechanisms, delivery methods, applications, benefits, and problems. ACS Biomater. Sci. Eng. 9 (3), 1190–1204. 10.1021/acsbiomaterials.2c01225 36779397

[B24] Huang H. Zhao G. Zhang Y. Xu J. Toth T. L. He X. (2017). Predehydration and ice seeding in the presence of trehalose enable cell cryopreservation. ACS Biomater. Sci. Eng. 3 (8), 1758–1768. 10.1021/acsbiomaterials.7b00201 28824959 PMC5558192

[B25] Jang T. H. Park S. C. Yang J. H. Kim J. Y. Seok J. H. Park U. S. (2017). Cryopreservation and its clinical applications. Integr. Med. Res. 6 (1), 12–18. 10.1016/j.imr.2016.12.001 28462139 PMC5395684

[B26] Janis B. R. Priddy M. C. Otto M. R. Kopechek J. A. Menze M. A. (2021). Sonoporation enables high-throughput loading of trehalose into red blood cells. Cryobiology 98, 73–79. 10.1016/j.cryobiol.2020.12.005 33359645

[B27] Joshi P. Ehrlich L. E. Gao Z. Bischof J. C. Rabin Y. (2022). Thermal analyses of nanowarming-assisted recovery of the heart from cryopreservation by vitrification. J. Heat. Transf. 144 (3), 031202. 10.1115/1.4053105 PMC8823202 35833152

[B28] Khosla K. Kangas J. Liu Y. Zhan L. Daly J. Hagedorn M. (2020). Cryopreservation and laser nanowarming of zebrafish embryos followed by hatching and spawning. Adv. Biosyst. 4 (11), e2000138. 10.1002/adbi.202000138 32996298 PMC8627598

[B29] Khosla K. Zhan L. Bhati A. Carley-Clopton A. Hagedorn M. Bischof J. (2019). Characterization of laser gold nanowarming: A platform for millimeter-scale cryopreservation. Langmuir 35 (23), 7364–7375. 10.1021/acs.langmuir.8b03011 30299961 PMC6536355

[B30] Kim H. J. Lee J. H. Hur Y. B. Lee C. W. Park S. H. Koo B. W. (2017). Marine antifreeze proteins: Structure, function, and application to cryopreservation as a potential cryoprotectant. Mar. Drugs 15 (2), 27. 10.3390/md15020027 28134801 PMC5334608

[B31] Kometas M. Christman G. M. Kramer J. Rhoton-Vlasak A. (2021). Methods of ovarian tissue cryopreservation: Is vitrification superior to slow freezing?-ovarian tissue freezing methods. Reprod. Sci. 28 (12), 3291–3302. 10.1007/s43032-021-00591-6 33939167

[B32] Lee S. Kim Y. M. Cheong H. T. Park C. K. Lee S. H. (2023). Effect of magnetized freezing extender on membrane damages, motility, and fertility of boar sperm following cryopreservation. Anim. (Basel) 13 (4), 634. 10.3390/ani13040634 PMC9951754 36830421

[B33] Lee Y. A. Kim Y. H. Kim B. J. Kim B. G. Kim K. J. Auh J. H. (2013). Cryopreservation in trehalose preserves functional capacity of murine spermatogonial stem cells. PLoS One 8 (1), e54889. 10.1371/journal.pone.0054889 23349986 PMC3551902

[B34] Li R. Hornberger K. Dutton J. R. Hubel A. (2020). Cryopreservation of human iPS cell aggregates in a DMSO-free solution-an optimization and comparative study. Front. Bioeng. Biotechnol. 8, 1. 10.3389/fbioe.2020.00001 32039188 PMC6987262

[B35] Liu X. Gao S. Niu Q. Zhu K. Ren L. Yuan X. (2022). Facilitating trehalose entry into hRBCs at 4 °C by alkylated ε-poly(l-lysine) for glycerol-free cryopreservation. J. Mater Chem. B 10 (7), 1042–1054. 10.1039/d1tb02674g 35080234

[B36] Liu X. Zhao G. Chen Z. Panhwar F. He X. (2018). Dual suppression effect of magnetic induction heating and microencapsulation on ice crystallization enables low-cryoprotectant vitrification of stem cell-alginate hydrogel constructs. ACS Appl. Mater Interfaces 10 (19), 16822–16835. 10.1021/acsami.8b04496 29688697 PMC6054798

[B37] Lovelock J. E. Bishop M. W. (1959). Prevention of freezing damage to living cells by dimethyl sulphoxide. Nature 183 (4672), 1394–1395. 10.1038/1831394a0 13657132

[B38] Lusianti R. E. Higgins A. Z. (2014). Continuous removal of glycerol from frozen-thawed red blood cells in a microfluidic membrane device. Biomicrofluidics 8 (5), 054124. 10.1063/1.4900675 25538811 PMC4224679

[B39] Manuchehrabadi N. Gao Z. Zhang J. Ring H. L. Shao Q. Liu F. (2017). Improved tissue cryopreservation using inductive heating of magnetic nanoparticles. Sci. Transl. Med. 9 (379), eaah4586. 10.1126/scitranslmed.aah4586 28251904 PMC5470364

[B40] Marquez-Curtis L. A. Janowska-Wieczorek A. McGann L. E. Elliott J. A. (2015). Mesenchymal stromal cells derived from various tissues: Biological, clinical and cryopreservation aspects. Cryobiology 71 (2), 181–197. 10.1016/j.cryobiol.2015.07.003 26186998

[B41] Mfarrej B. Bouchet G. Couquiaud J. Regimbaud L. Binninger S. Mercier M. (2017). Pre-clinical assessment of the Lovo device for dimethyl sulfoxide removal and cell concentration in thawed hematopoietic progenitor cell grafts. Cytotherapy 19 (12), 1501–1508. 10.1016/j.jcyt.2017.09.001 29037941

[B42] Miao S. Guo C. Jiang Z. Wei H. X. Jiang X. Gu J. (2022). Development of an open microfluidic platform for oocyte one-stop vitrification with cryotop method. Biosens. (Basel). 12 (9), 766. 10.3390/bios12090766 PMC9496857 36140151

[B43] Ntai A. La Spada A. De Blasio P. Biunno I. (2018). Trehalose to cryopreserve human pluripotent stem cells. Stem Cell. Res. 31, 102–112. 10.1016/j.scr.2018.07.021 30071393

[B44] Panhwar F. Chen Z. Hossain S. M. C. Wang M. Haider Z. Memon K. (2018). Near-infrared laser mediated modulation of ice crystallization by two-dimensional nanosheets enables high-survival recovery of biological cells from cryogenic temperatures. Nanoscale 10 (25), 11760–11774. 10.1039/c8nr01349g 29770427

[B45] Patra T. Pathak D. Gupta M. K. (2021). Strategies for cryopreservation of testicular cells and tissues in cancer and genetic diseases. Cell. Tissue Res. 385 (1), 1–19. 10.1007/s00441-021-03437-4 33791878

[B46] Pegg D. E. (2015). “Principles of cryopreservation,” in Cryopreservation and freeze-drying protocols. Editors Wolkers W. F. Oldenhof H. (Berlin, Germany: Springer), 3–19.

[B47] Piao Z. Patel M. Park J. K. Jeong B. (2022). Poly(l-alanine-co-l-lysine)-g-Trehalose as a biomimetic cryoprotectant for stem cells. Biomacromolecules 23 (5), 1995–2006. 10.1021/acs.biomac.1c01701 35412815

[B48] Rao W. Huang H. Wang H. Zhao S. Dumbleton J. Zhao G. (2015). Nanoparticle-mediated intracellular delivery enables cryopreservation of human adipose-derived stem cells using trehalose as the sole cryoprotectant. ACS Appl. Mater Interfaces 7 (8), 5017–5028. 10.1021/acsami.5b00655 25679454 PMC4734639

[B49] Schulz M. Risopatron J. Uribe P. Isachenko E. Isachenko V. Sanchez R. (2020). Human sperm vitrification: A scientific report. Andrology 8 (6), 1642–1650. 10.1111/andr.12847 32598551

[B50] Sharma A. Lee C. Y. Namsrai B. E. Han Z. Tobolt D. Rao J. S. (2023). Cryopreservation of whole rat livers by vitrification and nanowarming. Ann. Biomed. Eng. 51 (3), 566–577. 10.1007/s10439-022-03064-2 36183025 PMC10315167

[B51] Sharma A. Rao J. S. Han Z. Gangwar L. Namsrai B. Gao Z. (2021). Vitrification and nanowarming of kidneys. Adv. Sci. (Weinh). 8 (19), e2101691. 10.1002/advs.202101691 34382371 PMC8498880

[B52] Shu Z. Heimfeld S. Gao D. (2014). Hematopoietic SCT with cryopreserved grafts: Adverse reactions after transplantation and cryoprotectant removal before infusion. Bone Marrow Transpl. 49 (4), 469–476. 10.1038/bmt.2013.152 PMC4420483 24076548

[B53] Sreter J. A. Foxall T. L. Varga K. (2022). Intracellular and extracellular antifreeze protein significantly improves mammalian cell cryopreservation. Biomolecules 12 (5), 669. 10.3390/biom12050669 35625597 PMC9139014

[B54] Stefanic M. Ward K. Tawfik H. Seemann R. Baulin V. Guo Y. (2017). Apatite nanoparticles strongly improve red blood cell cryopreservation by mediating trehalose delivery via enhanced membrane permeation. Biomaterials 140, 138–149. 10.1016/j.biomaterials.2017.06.018 28649014

[B55] Stewart S. He X. (2019). Intracellular delivery of trehalose for cell banking. Langmuir 35 (23), 7414–7422. 10.1021/acs.langmuir.8b02015 30078320 PMC6382607

[B56] Stoll C. Holovati J. L. Acker J. P. Wolkers W. F. (2012). Synergistic effects of liposomes, trehalose, and hydroxyethyl starch for cryopreservation of human erythrocytes. Biotechnol. Prog. 28 (2), 364–371. 10.1002/btpr.1519 22275294

[B57] Stubbs C. Bailey T. L. Murray K. Gibson M. I. (2020). Polyampholytes as emerging macromolecular cryoprotectants. Biomacromolecules 21 (1), 7–17. 10.1021/acs.biomac.9b01053 31418266 PMC6960013

[B58] Syme R. Bewick M. Stewart D. Porter K. Chadderton T. Gluck S. (2004). The role of depletion of dimethyl sulfoxide before autografting: On hematologic recovery, side effects, and toxicity. Biol. Blood Marrow Transpl. 10 (2), 135–141. 10.1016/j.bbmt.2003.09.016 14750079

[B59] Taylor M. J. Weegman B. P. Baicu S. C. Giwa S. E. (2019). New approaches to cryopreservation of cells, tissues, and organs. Transfus. Med. Hemother 46 (3), 197–215. 10.1159/000499453 31244588 PMC6558330

[B60] Tomás R. M. F. Bailey T. L. Hasan M. Gibson M. I. (2019). Extracellular antifreeze protein significantly enhances the cryopreservation of cell monolayers. Biomacromolecules 20 (10), 3864–3872. 10.1021/acs.biomac.9b00951 31498594 PMC6794639

[B61] Verheijen M. Lienhard M. Schrooders Y. Clayton O. Nudischer R. Boerno S. (2019). DMSO induces drastic changes in human cellular processes and epigenetic landscape *in vitro* . Sci. Rep. 9 (1), 4641. 10.1038/s41598-019-40660-0 30874586 PMC6420634

[B62] Wang J. Zhao G. Zhang Z. Xu X. He X. (2016). Magnetic induction heating of superparamagnetic nanoparticles during rewarming augments the recovery of hUCM-MSCs cryopreserved by vitrification. Acta Biomater. 33, 264–274. 10.1016/j.actbio.2016.01.026 26802443 PMC5500173

[B63] Weng L. Beauchesne P. R. (2020). Dimethyl sulfoxide-free cryopreservation for cell therapy: A review. Cryobiology 94, 9–17. 10.1016/j.cryobiol.2020.03.012 32247742

[B64] Weng L. Stott S. L. Toner M. (2019). Exploring dynamics and structure of biomolecules, cryoprotectants, and water using molecular dynamics simulations: Implications for biostabilization and biopreservation. Annu. Rev. Biomed. Eng. 21, 1–31. 10.1146/annurev-bioeng-060418-052130 30525930 PMC8612073

[B65] Whaley D. Damyar K. Witek R. P. Mendoza A. Alexander M. Lakey J. R. (2021). Cryopreservation: An overview of principles and cell-specific considerations. Cell. Transpl. 30, 096368972199961. 10.1177/0963689721999617 PMC7995302 33757335

[B66] Wolkers W. F. Oldenhof H. (2015). Cryopreservation and freeze-drying protocols. Berlin, Germany: Springer.

[B67] Yao X. Jovevski J. J. Todd M. F. Xu R. Li Y. Wang J. (2020). Nanoparticle-Mediated intracellular protection of natural killer cells avoids cryoinjury and retains potent antitumor functions. Adv. Sci. (Weinh). 7 (9), 1902938. 10.1002/advs.201902938 32382476 PMC7201255

[B68] Yi X. Liu M. Luo Q. Zhuo H. Cao H. Wang J. (2017). Toxic effects of dimethyl sulfoxide on red blood cells, platelets, and vascular endothelial cells *in vitro* . FEBS Open Bio 7 (4), 485–494. 10.1002/2211-5463.12193 PMC5377396 28396834

[B69] Yong K. W. Laouar L. Elliott J. A. W. Jomha N. M. (2020). Review of non-permeating cryoprotectants as supplements for vitrification of mammalian tissues. Cryobiology 96, 1–11. 10.1016/j.cryobiol.2020.08.012 32910946

[B71] Zhan L. Han Z. Shao Q. Etheridge M. L. Hays T. Bischof J. C. (2022a). Rapid joule heating improves vitrification based cryopreservation. Nat. Commun. 13 (1), 6017. 10.1038/s41467-022-33546-9 36224179 PMC9556611

[B72] Zhan L. Rao J. S. Sethia N. Slama M. Q. Han Z. Tobolt D. (2022b). Pancreatic islet cryopreservation by vitrification achieves high viability, function, recovery and clinical scalability for transplantation. Nat. Med. 28 (4), 798–808. 10.1038/s41591-022-01718-1 35288694 PMC9018423

[B73] Zhang M. Oldenhof H. Sieme H. Wolkers W. F. (2016). Freezing-induced uptake of trehalose into mammalian cells facilitates cryopreservation. Biochim. Biophys. Acta 1858 (6), 1400–1409. 10.1016/j.bbamem.2016.03.020 27003129

[B74] Zhang Q. Liu B. Chong J. Ren L. Zhao Y. Yuan X. (2020). Combination of hydrophobically modified γ-poly(glutamic acid) and trehalose achieving high cryosurvival of RBCs. Sci. China Technol. Sci. 64 (4), 806–816. 10.1007/s11431-020-1549-2

[B75] Zhang Y. Wang H. Stewart S. Jiang B. Ou W. Zhao G. (2019). Cold-responsive nanoparticle enables intracellular delivery and rapid release of trehalose for organic-solvent-free cryopreservation. Nano Lett. 19 (12), 9051–9061. 10.1021/acs.nanolett.9b04109 31680526

[B76] Zhao G. Fu J. (2017). Microfluidics for cryopreservation. Biotechnol. Adv. 35 (2), 323–336. 10.1016/j.biotechadv.2017.01.006 28153517 PMC6236673

[B77] Zhou X. Liu Z. Shu Z. Ding W. Du P. Chung J. (2011). A dilution-filtration system for removing cryoprotective agents. J. Biomech. Eng. 133 (2), 021007. 10.1115/1.4003317 21280879

